# Effects of Dexmedetomidine on Emergence Agitation and Recovery Quality Among Children Undergoing Surgery Under General Anesthesia: A Meta-Analysis of Randomized Controlled Trials

**DOI:** 10.3389/fped.2020.580226

**Published:** 2020-11-13

**Authors:** Xiaoli Yang, Zhenyu Hu, Fei Peng, Guangxiang Chen, Yu Zhou, Qiange Yang, Xiaoling Yang, Maohua Wang

**Affiliations:** ^1^Department of Anesthesiology, The Affiliated Hospital of Southwest Medical University, Luzhou, China; ^2^Department of Radiology, The Affiliated Hospital of Southwest Medical University, Luzhou, China

**Keywords:** dexmedetomidine, emergence agitation, children, general anesthesia, meta-analysis

## Abstract

**Background:** Emergence agitation (EA) is one of the most common and intractable postoperative complications among children undergoing surgery under general anesthesia. Dexmedetomidine, an α(2)-adrenoceptor agonist, offers an ideal sedation, reduces preoperative anxiety, and facilitates smooth induction of anesthesia, and it is widely used in pediatric surgery. We aimed to evaluate the efficacy of dexmedetomidine for preventing emergence agitation in children after general anesthesia.

**Methods:** We comprehensively reviewed PubMed, Cochrane Library, EMBASE, and Web of Science databases to search all randomized controlled trials, published before April 22, 2020, investigating the efficacy of dexmedetomidine in preventing the emergence agitation in children after general anesthesia. The meta-analysis was performed using Review Manager 5.3. The primary outcome was the incidence of emergence agitation. Secondary outcomes included the number of patients requiring rescue analgesic, number of patients with postoperative nausea and vomiting, emergence time, extubation time, and time to discharge from the post-anesthesia care unit.

**Results:** We included a total of 33 studies, comprising 2,549 patients in this meta-analysis. Compared with saline, dexmedetomidine significantly reduced the emergence agitation incidence [risk ratio (RR) 0.29; 95% confidence interval (CI) 0.22–0.37; *p* < 0.00001], incidence of postoperative nausea and vomiting (RR 0.46; 95% CI 0.3–0.69; *p* = 0.0002), and the requirement of rescue analgesic (RR 0.29; 95% CI 0.18–0.44; *p* < 0.00001). Furthermore, children in the dexmedetomidine group experienced a longer emergence time [mean difference (MD) 2.18; 95% CI 0.81–3.56; *p* = 0.002] and extubation time (MD 0.77; 95% CI 0.22–1.31; *p* = 0.006) compared with those in the saline group. However, no significant difference was observed in the time to discharge from the post-anesthesia care unit (MD 2.22; 95% CI −2.29–6.74; *p* = 0.33) between the two groups. No significant differences were observed between the effects of dexmedetomidine and other drugs like midazolam, propofol, fentanyl, tramadol, and clonidine in terms of the emergence agitation incidence and other parameters, except for the requirement of rescue analgesic (RR 0.45; 95% CI 0.33–0.61; *p* < 0.00001).

**Conclusions:** Dexmedetomidine can prevent emergence agitation, relieves postoperative pain, decreases the requirement of rescue analgesic, and decreases the postoperative nausea and vomiting events.

## Introduction

Emergence agitation (EA) is a behavioral disturbance during the early post-anesthetic period, characterized by excitement, restlessness, disorientation, and other unusual behaviors, such as crying, shouting, kicking, inconsolability, and non-cooperation. EA incidence in children following sevoflurane anesthesia has been reported to be 10–80% (1, 2). EA is associated with the risk of self-harm, delayed discharge from the post-anesthesia care unit (PACU), extra burden on healthcare workers, increased parent dissatisfaction, and increased overall cost. Although the definition and criteria for EA are not clearly indicated, most children experiencing EA require a drug intervention to mitigate any threat to their safety. Patients with EA may unconsciously remove their endotracheal and stomach tubes, which can result in incision dehiscence, bleeding, urinary retention, and asphyxia. In addition, patients with EA often experience sympathetic excitation and instability of the circulatory system, which is dangerous if the patient is already having cardiovascular and cerebrovascular diseases. Several factors may cause EA, such as preschool age, preoperative anxiety, anesthetic, type of operation, and personal characteristics of the patient (3). Various drugs, including dexmedetomidine, midazolam, propofol, fentanyl, and melatonin have been investigated to prevent the EA incidence; however, the most favorable prophylactic treatment to decrease such incidence remains unknown. Among investigated drugs, dexmedetomidine is known as a highly selective α (2)-adrenoceptor agonist with sedative, anxiolytic, sympatholytic, and analgesic-sparing effects, which causes minimal depression of the respiratory function (4). The efficacy of dexmedetomidine toward EA prevention has been investigated in several clinical trials, using different administration routes and different dosages. We aimed to assess the effect of dexmedetomidine on EA incidence in the present study.

## Materials and Methods

This meta-analysis was conducted in accordance with the Preferred Reporting Items for Systematic Reviews and Meta-Analyses guidelines (5). The study was registered on PROSPERO (registration number: CRD42020187711).

### Criteria for Study Consideration

The trials selected for this meta-analysis met the following inclusion criteria:

- randomized controlled trials;
- children aged between 0 and 18 years;
- involving comparisons of dexmedetomidine as the intervention drug, delivered via intravenous or intranasal routes, with normal saline and other drugs (such as, midazolam, propofol, and fentanyl);
- published in the English language;
- involving EA assessment using evaluation scales, namely: five-point scale described by Cole, Aono four-point scale, Watcha four-point scale, a three-point scale, and Riker Sedation–Agitation Scale. Studies involving cardiac surgery were excluded.

### Outcome Measures

The primary outcome was EA incidence. Secondary outcomes included the number of patients requiring rescue analgesia, number of patients with postoperative nausea and vomiting (PONV), emergence time, extubation time, and time to discharge from the PACU.

### Search Strategy

PubMed, Cochrane Library, EMBASE, and Web of Science databases were comprehensively reviewed to identify randomized controlled clinical trials, published before April 22, 2020, investigating the efficacy of dexmedetomidine in EA prevention among children undergoing surgery with general anesthesia. In addition, the reference list of all the included studies was analyzed for additional potential publications. The detailed search strategies for each database are available in the Appendix.

### Data Extraction

Two experienced reviewers (Xiaoli Yang and Zhenyu Hu) independently screened the title and abstract of each literature to verify the suitability of the included trials. Data extraction was conducted independently by two reviewers using a standard data-collection form. Disagreements were resolved through discussion between the two reviewers and the corresponding author (Maohua Wang) to achieve a consensus. The following information was extracted from the included articles: primary author, publication year, country of the study, type of surgery, participant characteristics (age and sex), the administration route and dexmedetomidine dosage, control group's measure, scale, and criteria used for EA assessment.

### Risk-of-Bias Assessment

Two reviewers (Xiaoli Yang and Zhenyu Hu) independently assessed the quality of the included trials according to the Cochrane Collaboration tool (Cochrane, London, UK) (6). The included trials were scored as low risk, unclear, or high risk after assessment of bias under the following domains: random sequence generation (selection bias), allocation concealment (selection bias), blinding of participants and personnel (performance bias), blinding of outcome assessment (detection bias), incomplete outcome data (attrition bias), selective reporting (reporting bias), and other sources of bias. Disagreements were resolved through discussion between the two reviewers and the corresponding author.

### Data Analysis

The meta-analysis was conducted using Review Manager (Version 5.3, Nordic Cochrane Center). The Cochran Q test and Higgins *I*^2^ statistical tests were used to assess the statistical heterogeneity in the pooled results (7). *I*^2^ value was used to determine the level of heterogeneity in results; 0% ≤ *I*^2^ < 25% denoted no heterogeneity; 25% ≤ *I*^2^ < 50%, denoted low heterogeneity; 50% ≤ I^2^ < 75% denoted medium heterogeneity; and 75% ≤ *I*^2^ ≤ 100% denoted high heterogeneity. Data from all eligible RCTs were combined using the Mantel–Haenszel model to calculate the pooled risk ratio (RR) and 95% confidence interval (CI). Meta-analyses were performed using a random-effects model on account of clinical heterogeneity. This model provides an appropriate estimate of the average treatment effect when studies are statistically heterogeneous, and it typically yields relatively wide CIs resulting in a more conservative statistical claim. Statistical significance was set at a value of *p* < 0.05.

## Results

**Figure 1:**
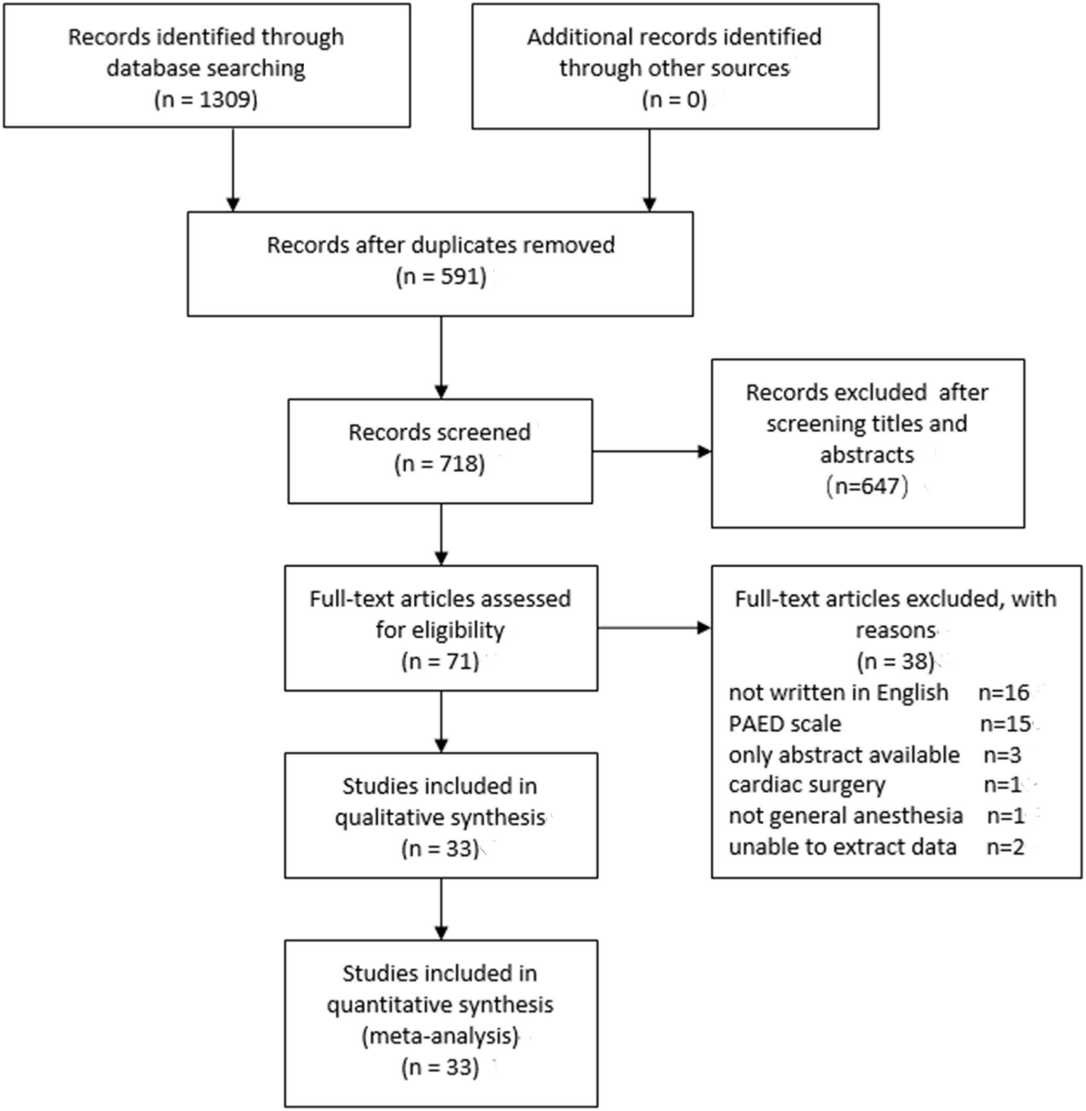
A PRISMA flow diagram of included/excluded studies.

We initially identified 1,309 relevant studies for the analysis, with 591 being excluded for duplication and 647 excluded after screening of title and abstract. Furthermore, 71 potentially eligible articles were reviewed for full texts, of which 36 were excluded for not satisfying the inclusion criteria. Moreover, we could not extract data from two studies. Finally, 33 (8–40) independent studies were included in the meta-analysis. Figure 1 presents the detailed selection process.

**Figure 2:**
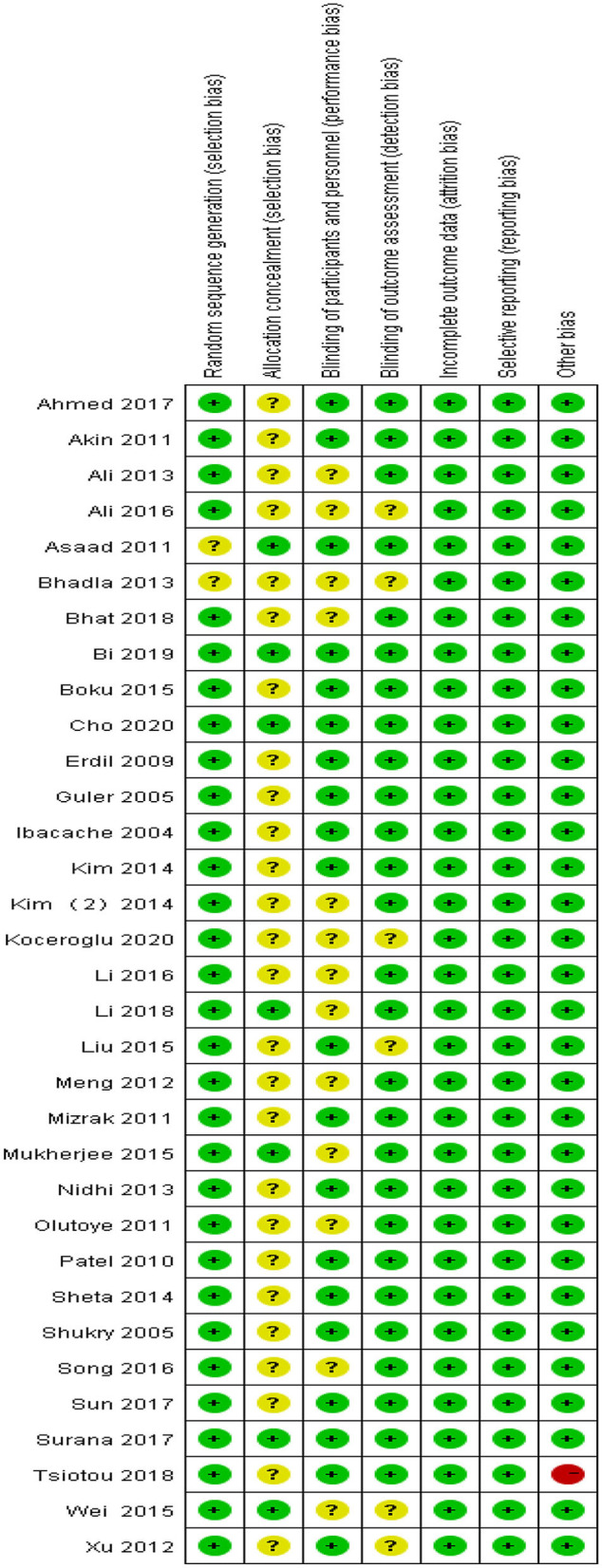
Risk-of-bias summary.

Table 1 summarizes the characteristics of the included trials. All the 33 included trials were published between 2004 and 2020. The sample size in the included trials ranged between 36 and 122, and a total of 2,549 subjects. Age of participants ranged from 3 months to 14 years. Figure 2 presents the risk of bias.

**Table 1:** Characteristics of included studies.

**Study ID**	**Age**	**Surgery type**	**Sample size**	**Anesthesia**	**Groups**	**Outcome**
Nidhi 2013 (12)	8–12 Y	Corrective surgery	36	Sev, N_2_O Intubation	DEX (9): 1 ug/kg iv followed by 0.5 ug/kg/h infusion; NS (9): volume-matched iv	ABCDE
Bi 2019 (36)	6–48 M	Fiberoptic bronchoscopy	40	Sev LMA	DEX (11):1 ug/kg intranasal; NS (11):0.01 ml/kg intranasal	ADE
Sun 2017 (30)	1–5 Y	Laparoscopic hernia repair	97	Sev LMA	DEX1(13):0.25 ug/kg iv; DEX2(14):0.5 ug/kg iv; DEX3(14):1 ug/kg iv; NS (15):2 ml iv	ADEF
Boku 2015 (37)	10–14 M	Palatoplasty	70	Sev, N_2_O Intubation	DEX (17):6 ug/kg/h for 10 min, followed by 0.4 ug/kg/h; NS (17): volume-matched iv	AD
Mizrak 2011 (18)	4.5–11 Y	Strabismus surgery	60	Ketamine Intubation	DEX (19):0.5 ug/kg iv; Placebo (19)	AC
Erdil 2009 (10)	2–7 Y	Adenoidectomy	90	Sev, N_2_O Intubation	DEX (19):0.5 ug/kg iv; Fentanyl (19):2.5 ug/kg iv; NS (19): iv	ADE
Guler 2005 (35)	3–7 Y	Adenotonsillectomy	60	Sev, N_2_O Intubation	DEX (19):0.5 ug/kg iv; NS (19): volume-matched iv	ABDE
Cho 2020 (13)	24 M−12 Y	Tonsillectomy	66	Sev Intubation	DEX (21):0.3 ug/kg iv; Midazolam (22):0.03 mg/kg iv	ABCDF
Li 2016 (23)	4–6 Y	Tonsillectomy	80	Des Intubation	DEX (24):0.2 ug/kg/h; NS (24): volume-matched iv	ABCDF
Wei 2015 (9)	3–24 M	Cleft palate repair	40	Sev, Propofol Remifentanil Intubation	DEX (11):0.8 ug/kg/min; NS (11): volume-matched iv	ABDE
Bhat 2018 (26)	1–8 Y	Inguinal hernia	90	Sev, N_2_O LMA	DEX1(19):0.5 ug/kg iv; DEX2(19):1 ug/kg iv; NS (19):5 ml iv	AEF
Ahmed 2017 (15)	3–7 Y	Tonsillectomy and/or Adenoidectomy	86	Sev Intubation	DEX (41):1 ug/kg intranasal; NS (41):1 ml intranasal	ABCDEF
Song 2016 (22)	2–6 Y	Strabismus surgery	103	Sev, N_2_O LMA	DEX1(14):0.25 ug/kg iv; DEX2(14):0.5 ug/kg iv; DEX3v (27):1 ug/kg iv; NS (14): iv	ACDF
Ali 2016 (11)	3–6 Y	Orthopedic surgery	90	Sev Intubation	DEX (19):0.3 ug/kg iv; Ketofol (19): ketamine 0.25 mg/kg and Propofol 1 mg/kg iv; NS (19):10 ml iv	AD
Mukherjee 2015 (19)	3–7 Y	Day care surgery	80	Sev Intubation	DEX (24):1 ug/kg intranasal; Clonidine (24):4 ug/kg intranasal	ABCDEF
Liu 2015 (27)	2–12 Y	Achilles-tendon lengthening	80	Sev Intubation	DEX (24):0.5 ug/kg iv; NS (24):10 ml iv	ADEF
Sheta 2014 (28)	3–6 Y	Dental rehabilitation	72	Sev, N_2_O Intubation	DEX (29):1 ug/kg intranasal; Midazolam (29):0.2 mg/kg	ABCE
Ali 2013 (8)	2–6 Y	Adenotonsillectomy	120	Sev, N_2_O Intubation	DEX (24):0.3 ug/kg iv; Propofol (24):1 mg/kg iv; NS (24):10 ml iv	ABDEF
Meng 2012 (31)	5–14 Y	Tonsillectomy	120	Sev Intubation	DEX1 (24):0.5 ug/kg iv followed by 0.2 ug/kg/h; DEX2 (24):1 ug/kg followed by 0.4 ug/kg/h; lactated Ringer (24): iv	ADEF
Xu 2012 (32)	3–7 Y	Vitreoretinal surgery	60	Sev, Remifentanil Intubation	DEX (19):0.5 ug/kg, iv; NS (19):10 ml iv	ADE
Asaad 2011 (33)	5–10 Y	Elective surgery	88	Sev, N_2_O Intubation	DEX (19):0.15 ug/kg, iv; Fentanyl (27):1 ug/kg; NS (19):10 ml iv	AEF
Ibacache 2004 (14)	1–10 Y	Inguinal hernia repair, orchiopexy, or circumcision	90	Sev, N_2_O LMA	DEX1(19):0.15 ug/kg, iv; DEX2(19):1 ug/kg; NS (19):10 ml iv	AEF
Olutoye 2011 (34)	3–12 Y	Tonsillectomy and adenoidectomy	109	Sev, N_2_O Intubation	DEX1(23):0.75 ug/kg, iv; DEX2(32):1 ug/kg,iv;Morphine1(19):50 ug/kg iv; Morphine2(23):100 ug/kg iv	AB
Li 2018 (25)	2–7 Y	Adenoidectomy with or without tonsillectomy	90	Propofol, Remifentanil Intubation	DEX1(19):1 ug/kg, iv; DEX2(19):2 ug/kg, iv; NS (19):1 ml iv	A
Kim 2014 (16)	1–5 Y	Strabismus Surgery	94	Des Intubation	DEX (42):0.2 ug/kg/h; NS (42): iv	ACDEF
Patel 2010 (20)	2–10 Y	Tonsillectomy and adenoidectomy	122	Sev, N_2_O Intubation	DEX (61):2 ug/kg followed by 0.7 ug/kg/h; Fentanyl (61):1 ug/kg iv	ACDE
Koceroglu 2020 (21)	2–9 Y	Adenotonsillectomy	60	Sev, N_2_O Intubation	DEX (19):1 ug/kg iv; Tramadol (19):1.5 mg/kg iv	ABDE
Akin 2011 (17)	2–9 Y	Adenotonsillectomy	90	Sev, N_2_O Intubation	DEX (43):1 ug/kg intranasal; Midazolam (43):0.2 mg/kg intranasal	ABCD
Kim 2014 (38)	1–5 Y	Hernioplasty or orchiopexy	40	Sev LMA	DEX (11):1 ug/kg followed by 0.1 ug/kg/h; NS (11): same amount IV	AD
Shukry 2005 (39)	1–10 Y	Outpatient surgery	46	Sev Intubation	DEX (13):0.2 ug/kg/h infusion; NS (13): volume-matched iv	ADF
Surana 2017 (40)	6 M-12 Y	Cleft palate surgery	60	Iso, N_2_O Intubation	DEX (19):1 ug/kg followed by 0.5 ug/kg/h; Midazolam (19):0.05 mg/kg iv	ACDE
Tsiotou 2018 (24)	3–14 Y	Tonsillectomy	60	Propofol, Remifentanil Intubation	DEX (28):1 ug/kg iv; NS (31):50 ml iv	ACD
Bhadla 2013 (29)	5–12 Y	Ophthalmic day-care surgery	60	Iso Intubation	DEX (19):0.4 ug/kg iv; Midazolam (19):0.04 mg/kg iv	A

*A, emergence agitation; B, PONV; C, requiring rescue anesthetic; D, extubation time; E, emergence time; F, Time to discharge from the PACU; M, months; Y, years; DEX, dexmedetomidine; NS, normal saline; LMA, laryngeal mask airway; Sev, sevoflurane; Des, desflurane; Iso, isoflurane*.

### Primary Outcome

**Figure 3:**
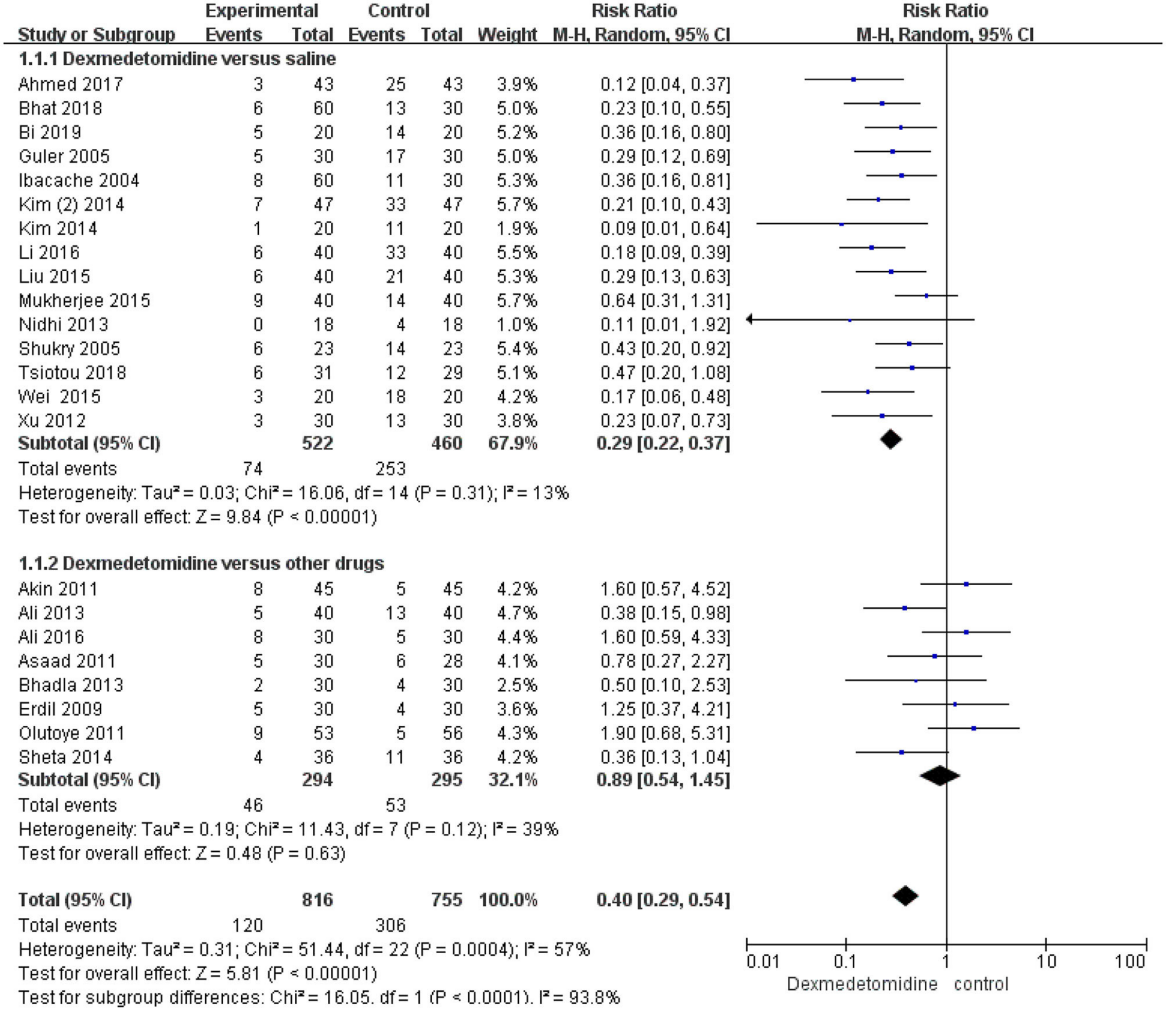
Incidence of emergence agitation (EA): dexmedetomidine vs. saline and other drugs. Forest plot shows that pooled trials were in favor of dexmedetomidine when compared to saline; there was no significant difference between dexmedetomidine and other drugs.

EA incidence was reported in all the included studies. The results of EA in 10 studies could not be pooled. Among the included trials, 15 studies compared the efficacy of dexmedetomidine with that of saline in preventing EA incidence, and the total number of reported events in these trials was 327. The reported EA incidences in the included trials were 14.2% (74 out of 522) and 55% (253 out of 460) in the dexmedetomidine and saline groups, respectively. Dexmedetomidine was associated with a significant reduction in the EA incidence, compared with saline (RR 0.29; 95% CI 0.22–0.37; *p* < 0.00001) (Figure 3), and heterogeneity was not observed (*I*^2^ = 13%). Compared with other anesthetics, dexmedetomidine was not found to significantly reduce the EA incidence (RR 0.89; 95% CI 0.54–1.45; *p* = 0.63; *I*^2^ = 39%) (Figure 3).

**Figure 4:**
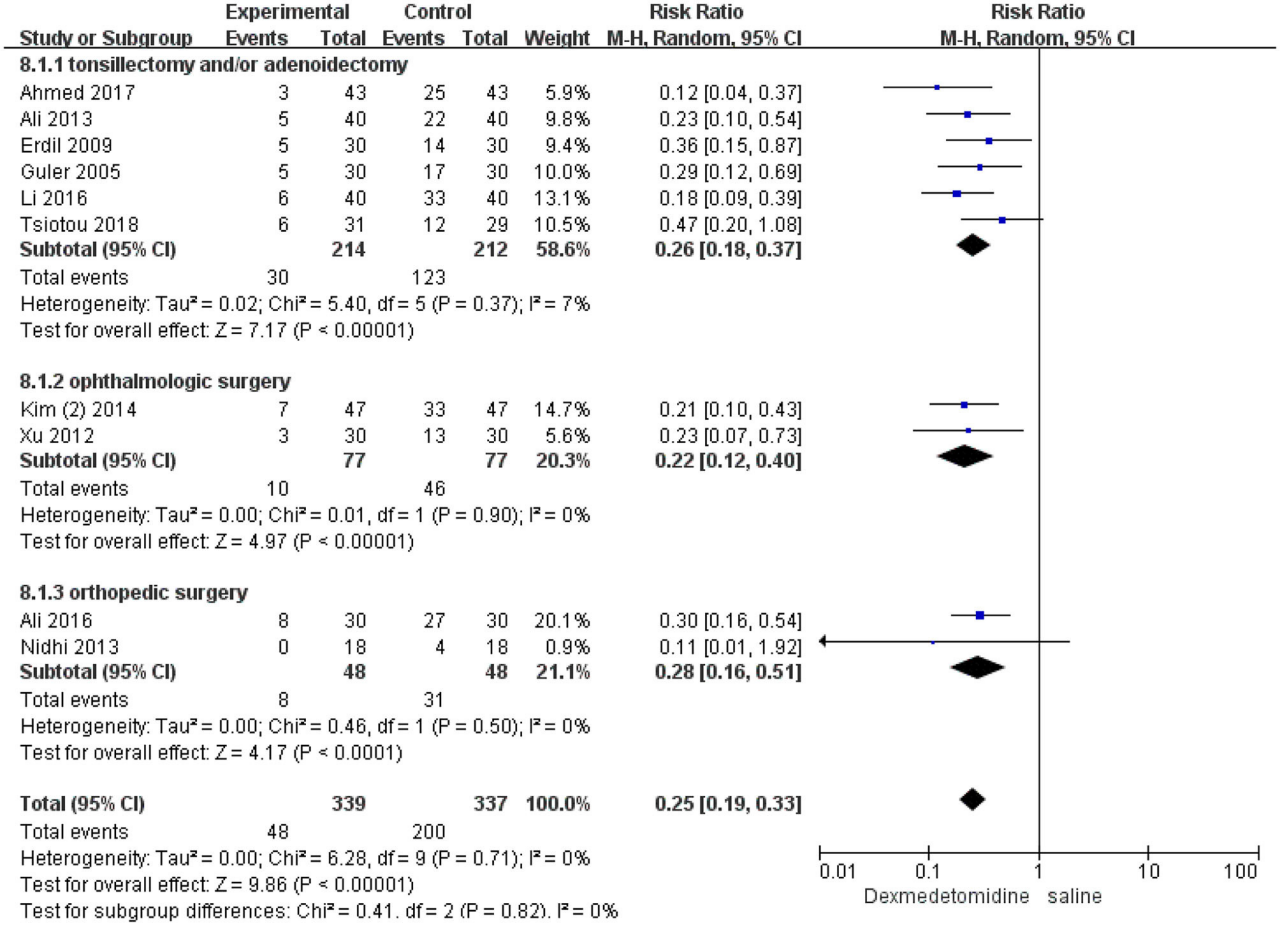
Incidence of EA in different surgeries: dexmedetomidine vs. saline.

**Figure 5:**
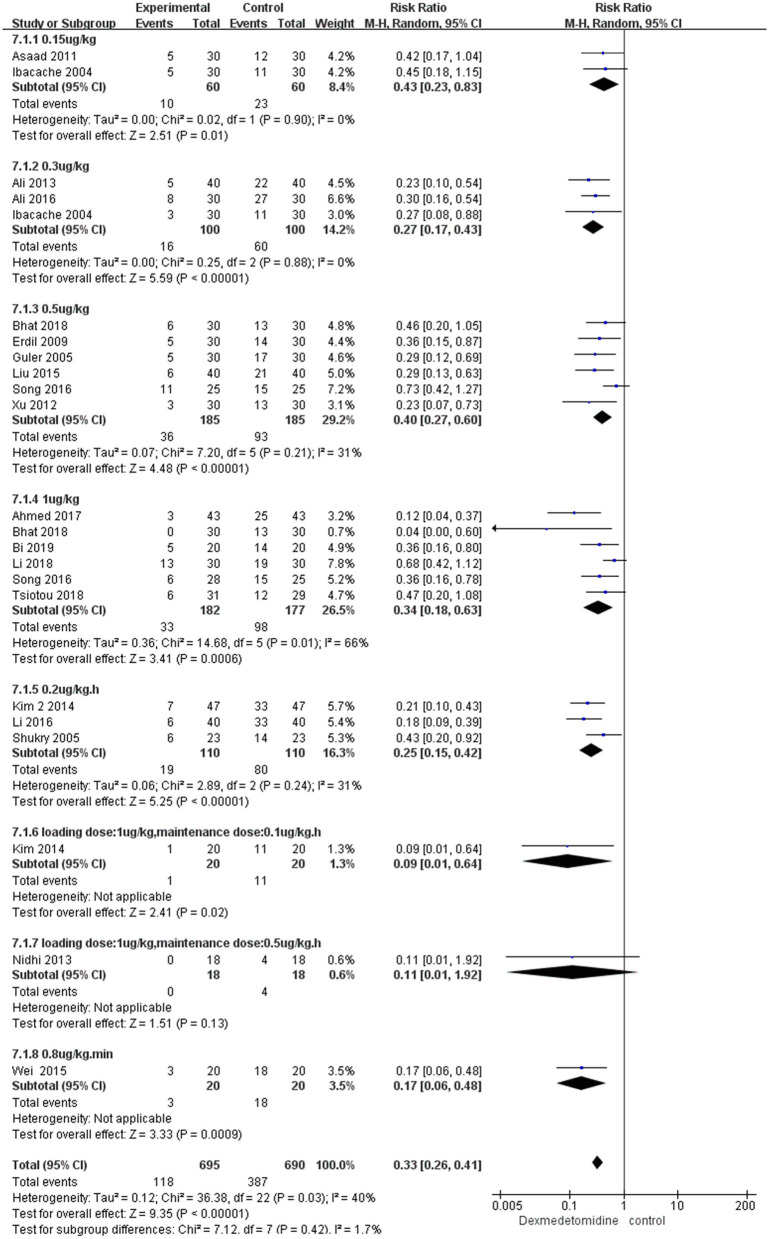
Incidence of EA in different dosages of dexmedetomidine vs. saline.

**Figure 6:**
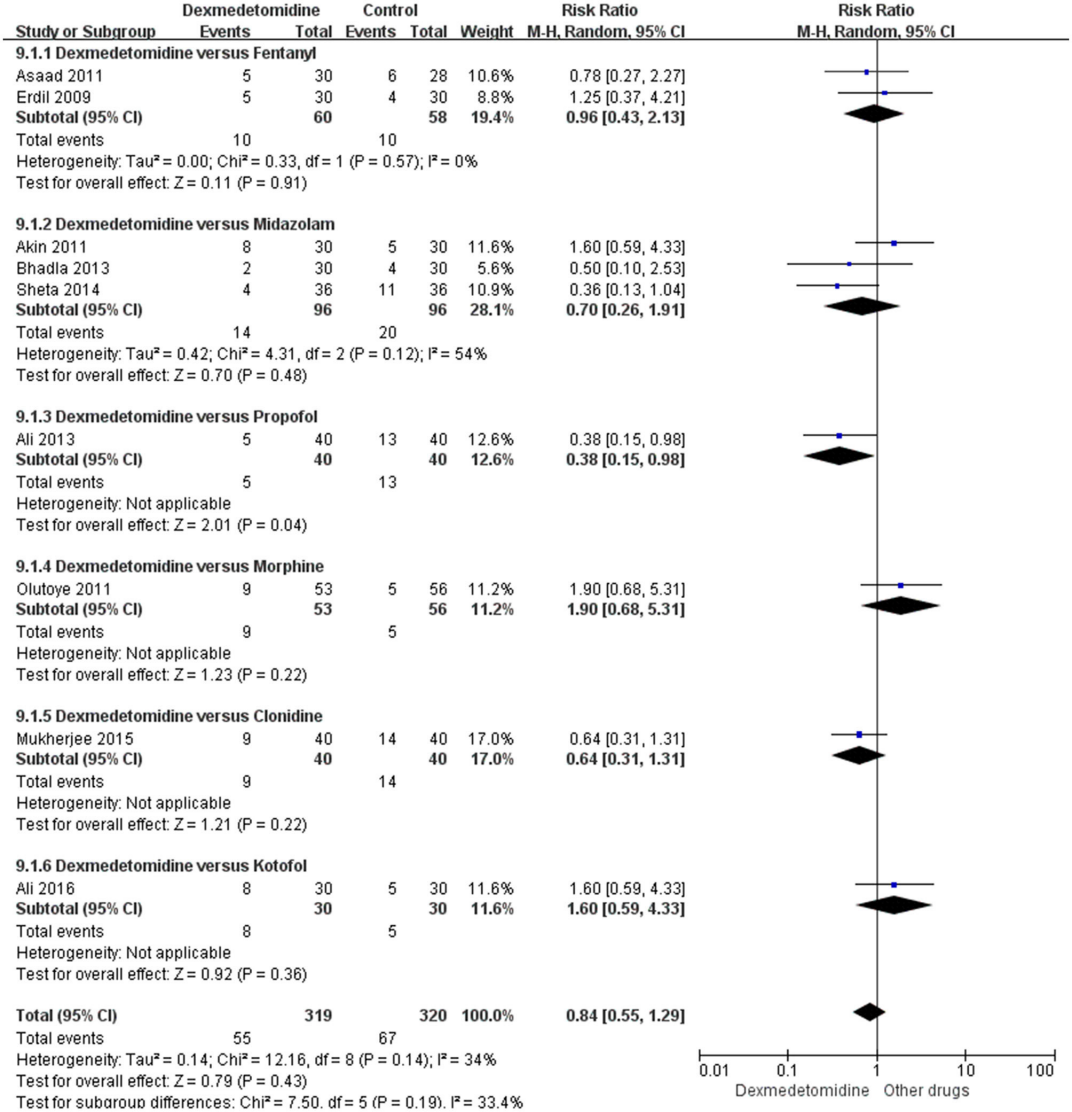
Forest plot for incidence of EA in dexmedetomidine vs. every other drug.

In the subgroup analyses, six studies indicated that dexmedetomidine significantly reduces the EA incidence (RR 0.26; 95% CI 0.18–0.37; *p* < 0.00001; *I*^2^ = 7%) in adenoidectomy with or without tonsillectomy compared with saline. In addition, two studies indicated that dexmedetomidine reduces the EA incidence in ophthalmologic surgery (RR 0.22; 95% CI 0.12–0.4; *p* < 0.00001; *I*^2^ = 0%) and orthopedic surgery (RR 0.28; 95% CI 0.16–0.51; *p* < 0.0001; *I*^2^ = 0%) compared with saline, respectively (Figure 4). Moreover, 16 studies, different dosages of dexmedetomidine with that of saline, indicated that each dosage of dexmedetomidine is effective in preventing EA (Figure 5). Finally, two studies compared dexmedetomidine and fentanyl, three studies compared dexmedetomidine and midazolam, and one study compared dexmedetomidine and propofol; morphine, clonidine, kotofol, respectively, showed no significant differences between them in EA incidence (*p* > 0.05) (Figure 6).

### Secondary Outcomes

**Figure 7:**
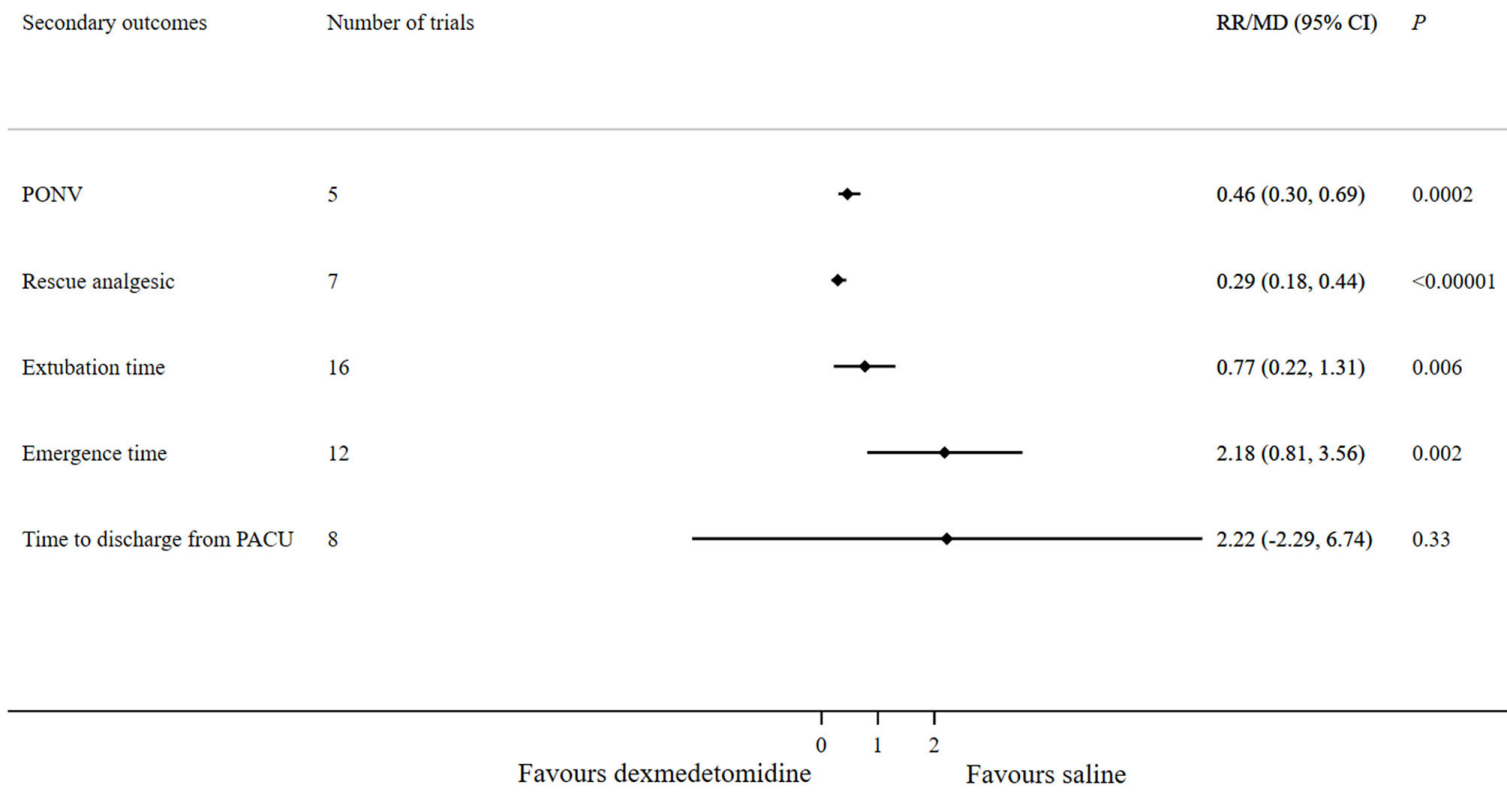
Forest plot for secondary outcomes (dexmedetomidine vs. saline).

Compared with saline, dexmedetomidine significantly reduced the PONV incidence (RR 0.46; 95% CI 0.3–0.69; *p* = 0.0002) and the requirement of rescue analgesic (RR 0.29; 95% CI 0.18–0.44; *p* < 0.00001). Extubation time (MD 0.77; 95% CI 0.22–1.31; *p* = 0.006) and emergence time (MD 2.18; 95% CI 0.81–3.56; *p* = 0.002) were longer in the dexmedetomidine group compared with the saline group. Eight studies assessing the time to discharge from the PACU reported no significant difference between the dexmedetomidine and saline groups (MD 2.22; 95% CI −2.29–6.74; *p* = 0.33) (Figure 7).

**Figure 8:**
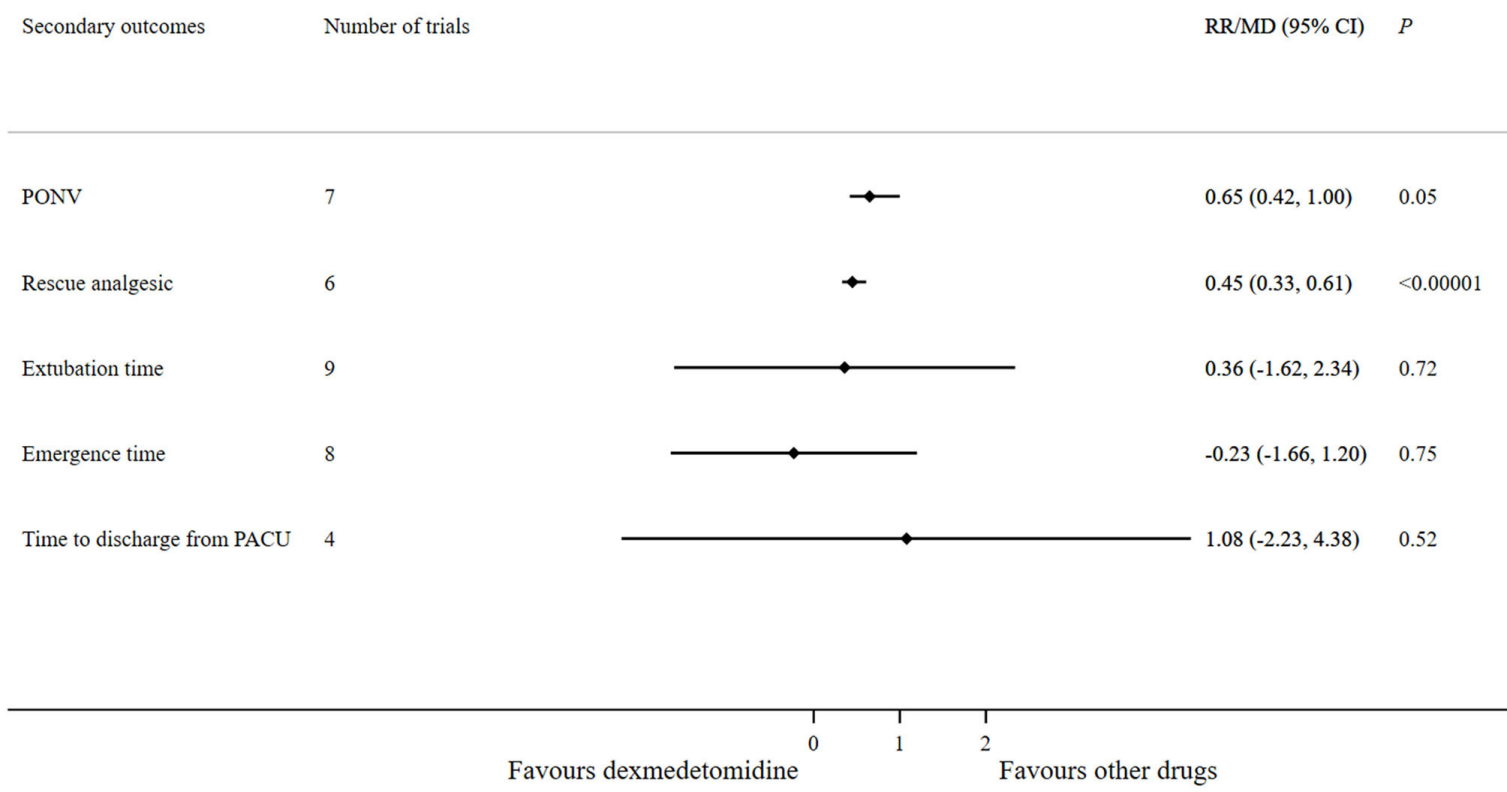
Forest plot for secondary outcomes (dexmedetomidine vs. other drugs).

Compared with other anesthetics, dexmedetomidine significantly reduced the requirement of rescue analgesic (RR 0.45; 95% CI 0.33–0.61; *p* < 0.00001). However, no significant differences were observed between the dexmedetomidine and other anesthetics in terms of PONV incidence (RR 0.65; 95% CI 0.42–1.00; *p* = 0.05), extubation time (MD 0.36; 95% CI −1.62–2.34; *p* = 0.72), emergence time (MD −0.23; 95% CI −1.66–1.2; *p* = 0.75), and time to discharge from the PACU (MD 1.08; 95% CI −2.23–4.38; *p* = 0.52) (Figure 8).

### Publication Bias

**Figure 9:**
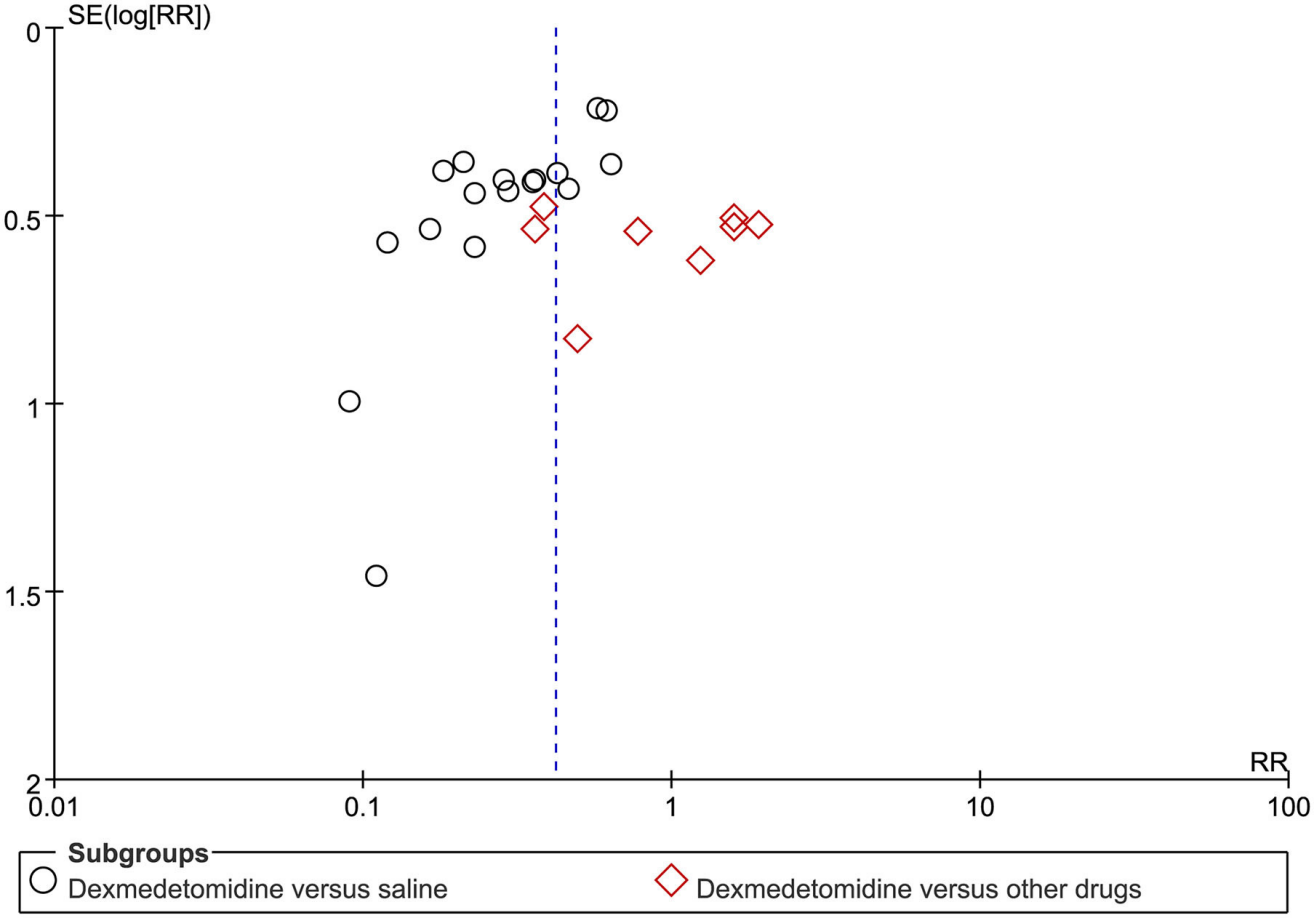
Funnel plot for evaluation of potential publication bias.

Funnel plots for the primary outcome indicated a slight publication bias (Figure 9).

## Discussion

This meta-analysis indicates that dexmedetomidine is efficient in preventing EA, avoiding PONV, and alleviating pain in children under general anesthesia compared with saline, although with prolonged emergence time and extubation time. However, time to discharge from PACU was similar in patients after anesthesia with saline and other anesthetics.

EA is one of the most common postoperative complications in pediatric surgery, following general anesthesia. Although many drugs have been applied to prevent EA, consensus on the most effective drug is lacking.

Several studies comparing the efficacy of dexmedetomidine with that of placebo have been published (41, 44, 45). In line with these studies, this meta-analysis demonstrated that compared with saline, intranasal or intravenous administration of dexmedetomidine significantly reduces the EA incidence. Subgroup analyses with different dosages and different operations indicated that each dosage of dexmedetomidine is efficient in preventing EA, compared with saline. Since the optimal dosage of dexmedetomidine for preventing EA could not be deduced from the present analysis, the lowest dose according to the patients' physical condition and operation type can be considered to avoid the side effects of dexmedetomidine. More prospective studies comparing the effects of different dosages of dexmedetomidine on EA are required to establish the optimal dose. Dexmedetomidine used in tonsillectomy, adenoidectomy, ophthalmologic, and orthopedic surgeries lower the EA incidence compared with saline, which is consistent with the findings of Cho et al. (43), Jiao et al. (46), and Tan et al. (42). Tonsillectomy with or without adenoidectomy is commonly associated with throat pain and discomfort, and the EA risk associated with this procedure is up to 55.88% and may involve “a sense of suffocation” because of edema, difficulty in swallowing, and nausea. Dexmedetomidine, through adequate analgesia and sedation, significantly decreases the EA occurrence, and can be widely used in suitable patients. Dexmedetomidine can reduce EA incidence not only in children receiving general anesthesia but also in children undergoing magnetic resonance imaging, without hemodynamic or respiratory distress that prolong the time to discharge from the hospital (47). This meta-analysis indicates that the PONV event is reduced in children following dexmedetomidine anesthesia compared with that in children under saline administration; however, a meta-analysis published in 2014 could not establish the efficacy of dexmedetomidine in lowering the PONV incidence (45). Dexmedetomidine was also found to significantly reduce the requirement of rescue analgesic, which is consistent with the findings of Cho et al. (43) and Jun et al. (48). Inadequate analgesia is one of the factors contributing to postoperative agitation. Dexmedetomidine activates the α (2)-adrenergic receptor located in the presynaptic and posterior membranes of the spinal cord and inhibits the peripheral nerve fibers A and C which may contribute to the decrease in the demand for a rescue analgesic. Time to emergence and extubation were longer in the dexmedetomidine group compared with saline groups; heterogeneity was observed, may have originated from the study of Yang, wherein children with cerebral palsy were included, and dexmedetomidine reduced the sevoflurane mandate during surgery, thereby decreasing the emergence time and extubation time, which contradict the findings of other studies (27). Unexpectedly, no significant difference was observed in the time to discharge from the PACU between the dexmedetomidine and saline groups; heterogeneity was observed when these studies were pooled owing to the study by Bhat. Overall, we found that dexmedetomidine slightly increases the time to discharge from the PACU by 2.22 min, relative to saline, which is shorter than what was reported in a study by Ni et al. Dexmedetomidine offers favorable analgesia and sedation, and may avoid restlessness, unusual behaviors, such as kicking, shouting, and crying in children, which might account for the reduced stay time in the PACU. Generally, dexmedetomidine is effective in preventing EA, without prolonging the time to discharge from the PACU, and thus, it could decrease the burden on healthcare workers and parents.

Except for the requirement of rescue analgesic, no significant differences were observed between the dexmedetomidine and other anesthetic groups in terms of the EA incidence, PONV event, emergence time, extubation time, and time to discharge from the PACU, which is in line with the findings of Feng et al. (49) and Peng et al. (50). Midazolam, a γ-amino-butyric acid receptor inhibitor, is commonly used for premedication in children, which provides effective sedation, anxiolytic effect, and anterograde amnesia; however, it also produces side effects, such as postoperative behavioral changes, cognitive impairment, paradoxical reactions, and respiratory depression (17, 28, 29). Unlike midazolam, dexmedetomidine exerts its hypnotic action through the activation of central pre- and post-synaptic α(1)-adrenergic receptors in the locus coeruleus, rather than the cerebral cortex, and induces a natural sleep status in which the patients remain easily arousable and cooperative, and therefore, it is increasingly used in children (4). Fentanyl, a short-acting opioid analgesic, also produces sedative effects. All the three drugs act on different sites to exert sedative and analgesic effects. Although we observed no significant difference between the effects of these drugs, we recommend that dexmedetomidine is the most suitable option for EA prophylaxis in children as a premedicant because of fewer adverse effects.

### Limitations

This meta-analysis has some limitations. First, studies comparing the efficacy of dexmedetomidine with that of midazolam, fentanyl, clonidine, tramadol, and ketofol are limited; a stronger evidence is required to confirm the effectiveness of dexmedetomidine in preventing EA relative to the above drugs. Second, the patients' age in the included studies was variable, which might have caused discrepancy in the results because pharmacokinetics and pharmacodynamics vary between the age of 3 months and 14 years, which may lead to different results. Third, heterogeneity was observed in some analyses such as in the emergence time, extubation time, and time to discharge from the PACU; however, sensitivity analysis demonstrated that the change in total effects is independent of the inclusion or exclusion of trials.

## Conclusions

Compared with saline, dexmedetomidine decreases the EA risk, PONV incidence, and requirement of rescue analgesic in children undergoing surgery under general anesthesia. Overall, dexmedetomidine is an excellent choice to prevent EA, compared with other drugs.

## Data Availability Statement

All datasets generated for this study are included in the article/Supplementary Material.

## Author Contributions

All authors listed have made a substantial, direct and intellectual contribution to the work, and approved it for publication.

## Conflict of Interest

The authors declare that the research was conducted in the absence of any commercial or financial relationships that could be construed as a potential conflict of interest.
