# Supplementary figures and images for: Effects of Dexmedetomidine on Emergence Agitation and Recovery Quality Among Children Undergoing Surgery Under General Anesthesia: A Meta-Analysis of Randomized Controlled Trials
Source: Front Pediatr. 2020 Nov 13;8:580226. doi: 10.3389/fped.2020.580226 (PMC7694572; doi:10.3389/fped.2020.580226)

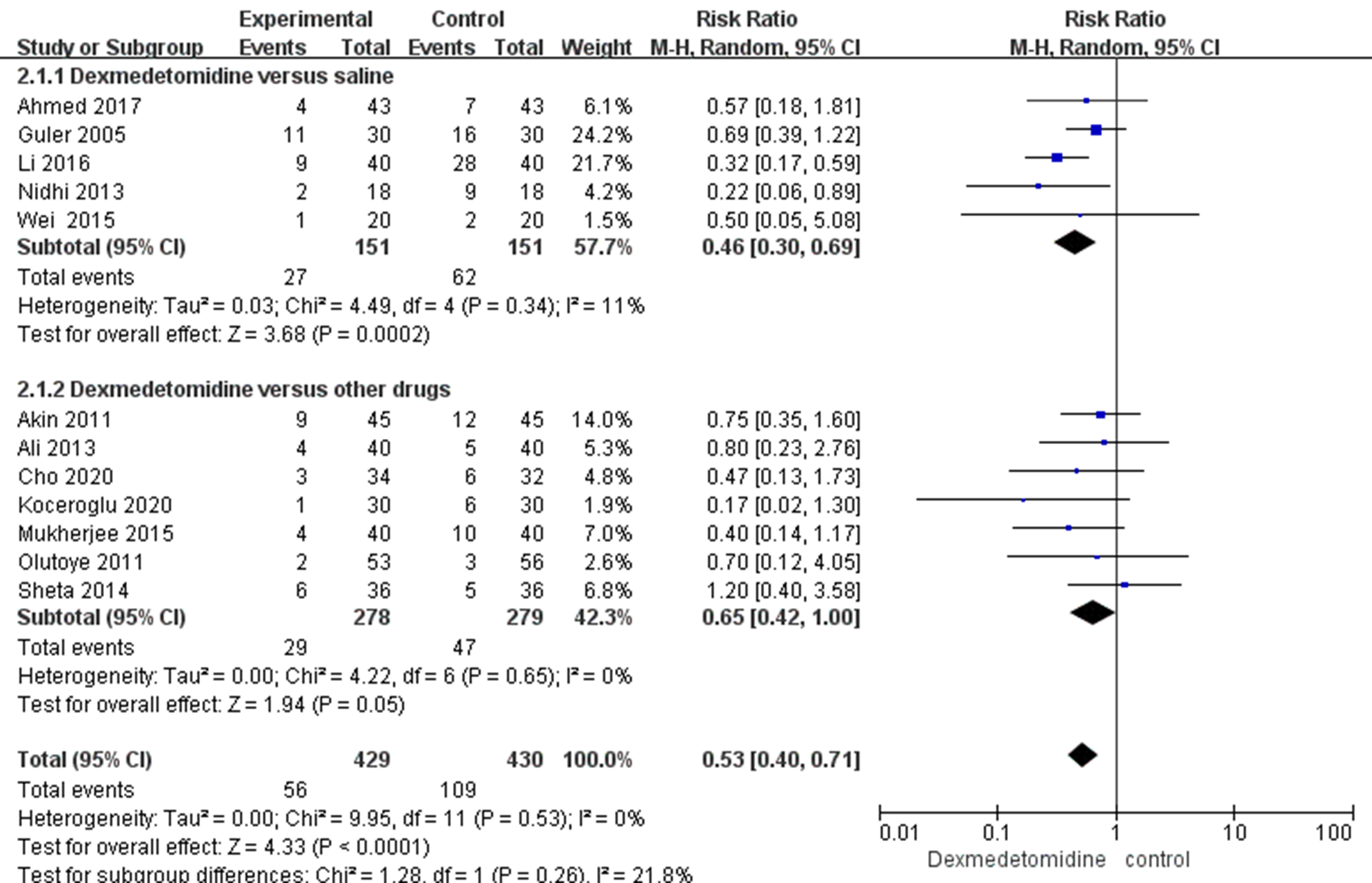

Supplement: Supplementary file 1 [file Data_Sheet_1.zip › Supplementary Material/Supplementary Figure 1.tif]

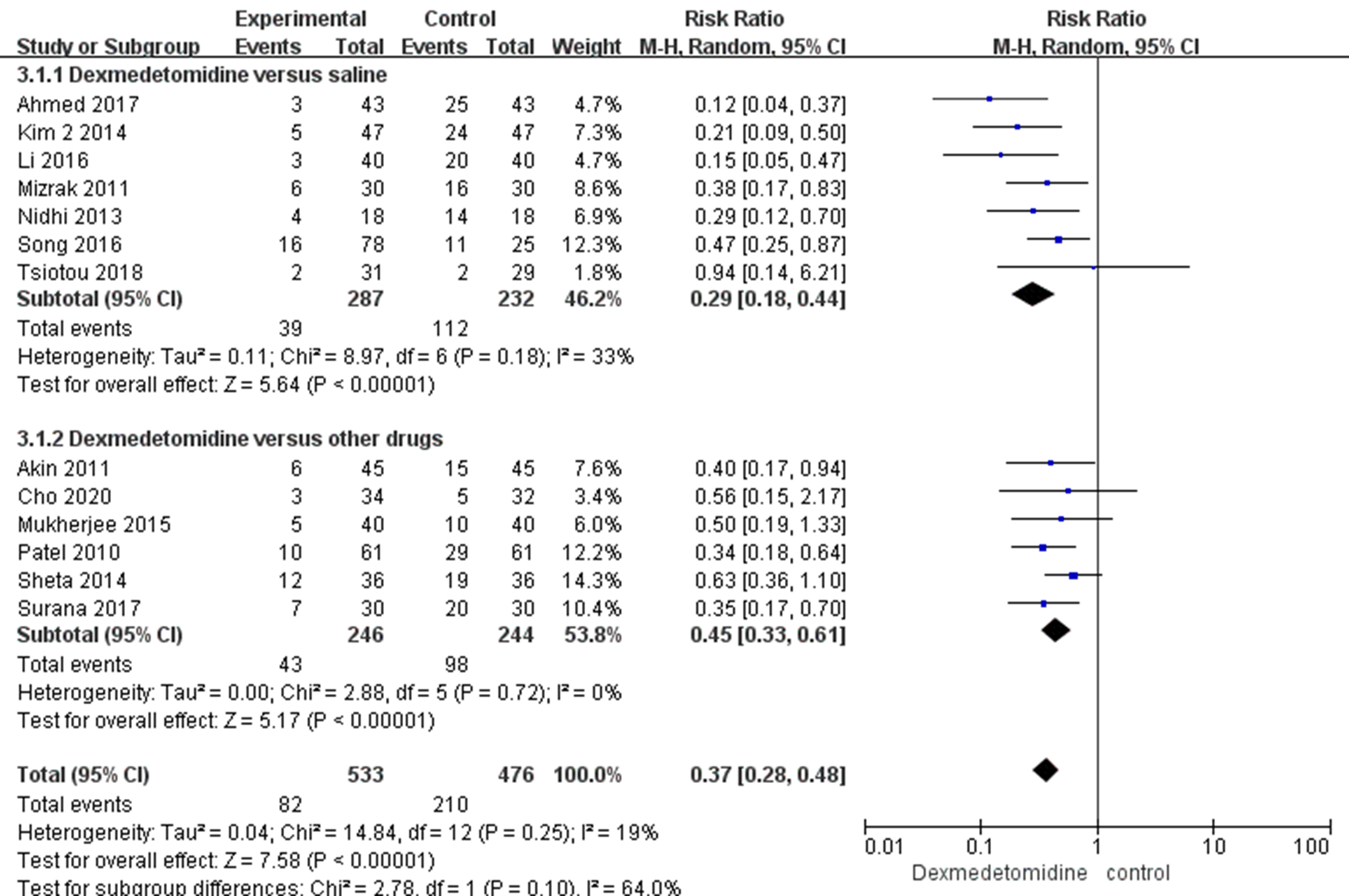

Supplement: Supplementary file 1 [file Data_Sheet_1.zip › Supplementary Material/Supplementary Figure 2.tif]

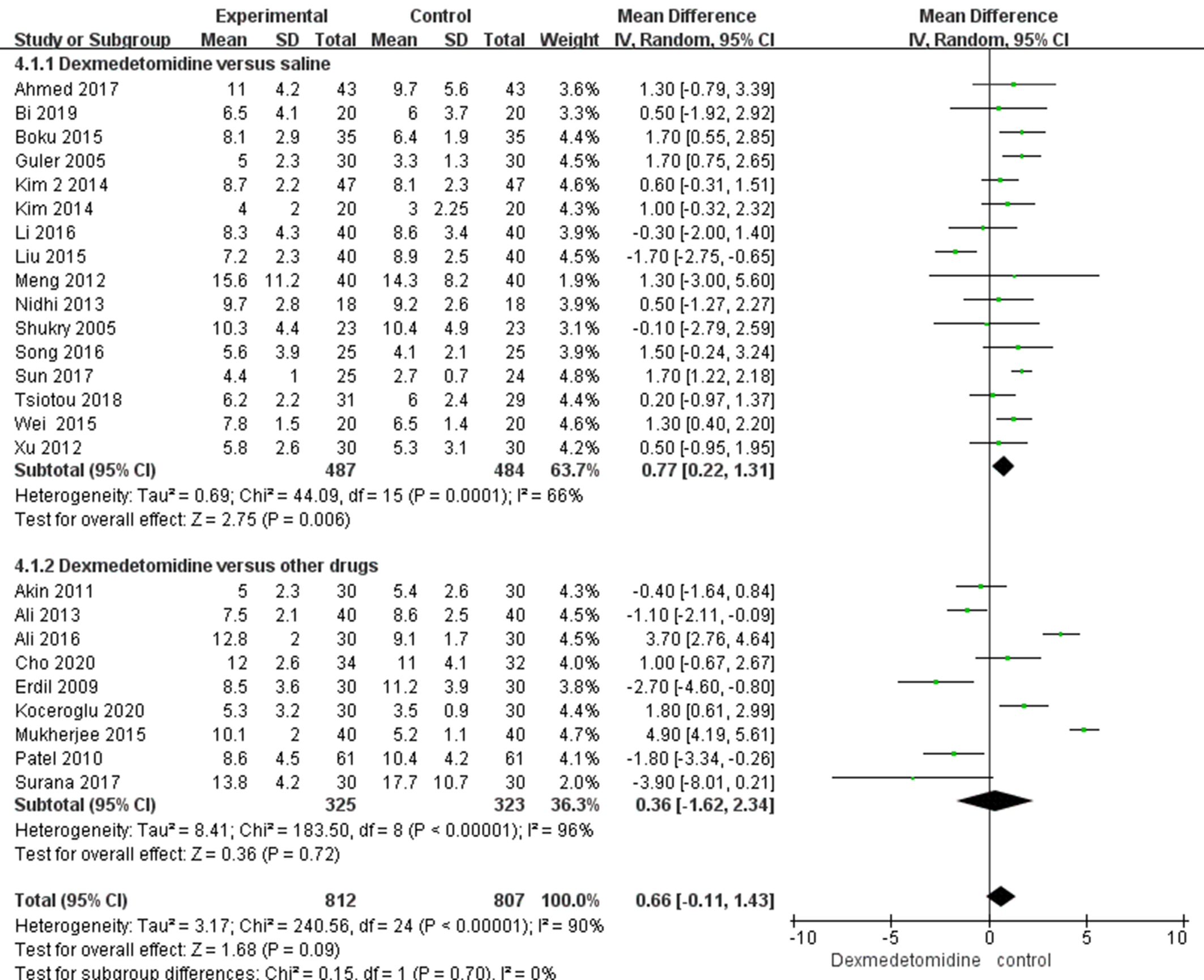

Supplement: Supplementary file 1 [file Data_Sheet_1.zip › Supplementary Material/Supplementary Figure 3.tif]

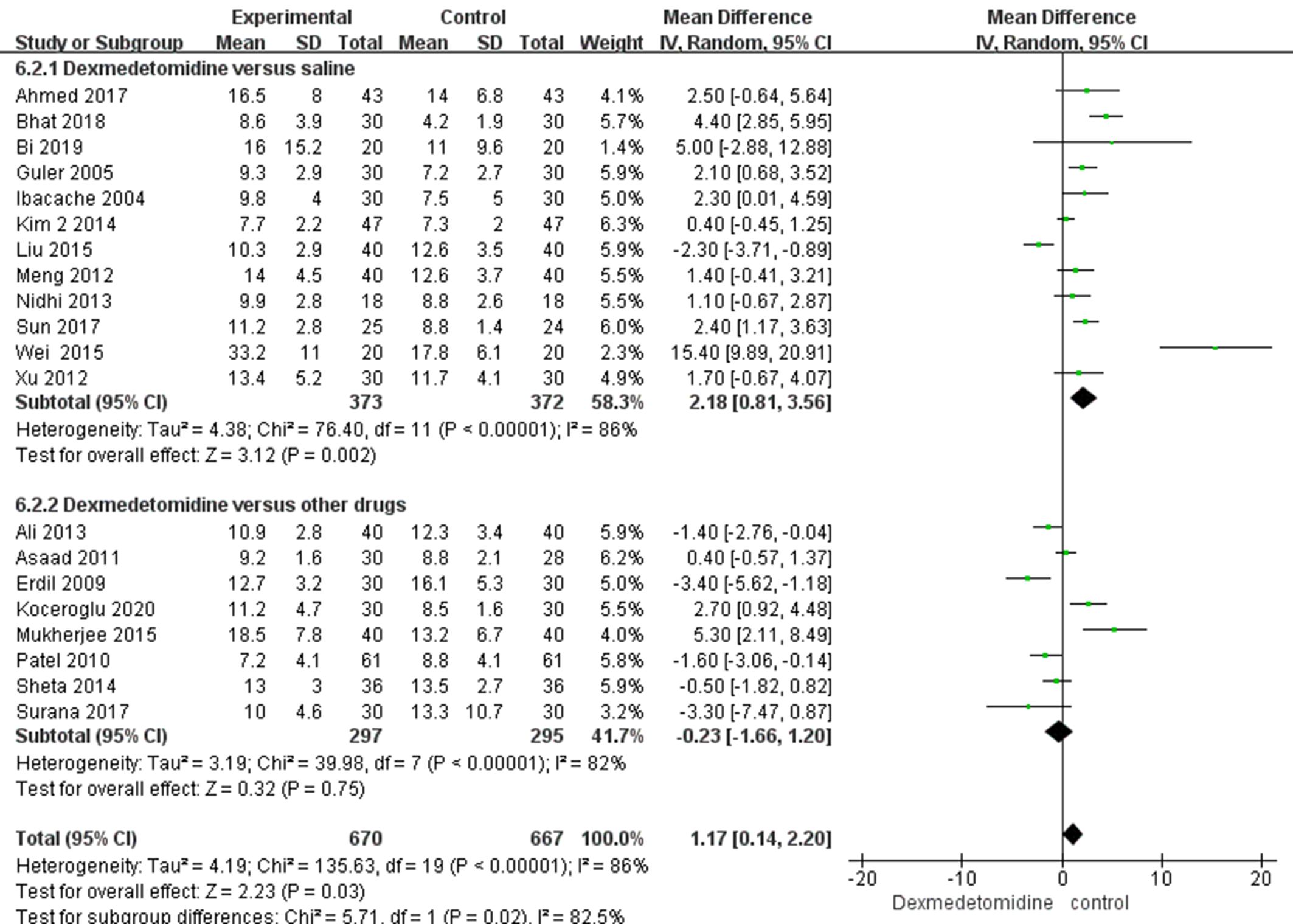

Supplement: Supplementary file 1 [file Data_Sheet_1.zip › Supplementary Material/Supplementary Figure 4.tif]

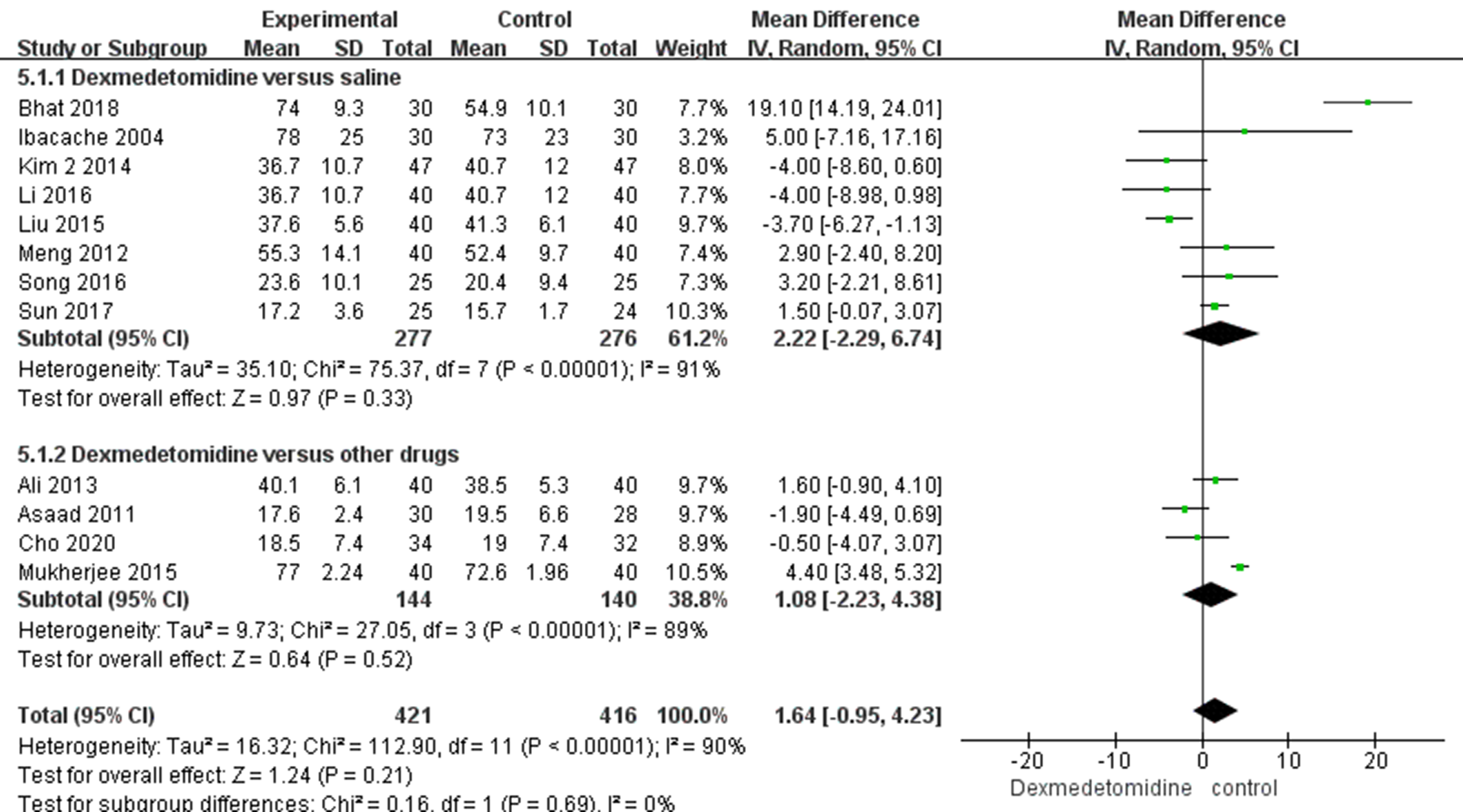

Supplement: Supplementary file 1 [file Data_Sheet_1.zip › Supplementary Material/Supplementary Figure 5.tif]
