# Supplementary material for: Effects of Dexmedetomidine on Emergence Agitation and Recovery Quality Among Children Undergoing Surgery Under General Anesthesia: A Meta-Analysis of Randomized Controlled Trials
Source: Front Pediatr. 2020 Nov 13;8:580226. doi: 10.3389/fped.2020.580226 (PMC7694572; doi:10.3389/fped.2020.580226)
Supplement: Supplementary file 1 [file Data_Sheet_1.zip › Supplementary Material/Appendix.doc]

Search strategies

| **Pubmed 218** | #1 Dexmedetomidine [Mesh]  #2 Dexmedetomidin*[Title/Abstract] OR Precedex [Title/Abstract] OR MPV1440[Title/Abstract] OR MPV 1440[Title/Abstract] OR MPV-1440[Title/Abstract]  #3 #1 OR #2  #4 Emergence Delirium [Mesh]  #5 (emergence*[Title/Abstract] OR postoperative [Title/Abstract] OR postanesthetic [Title/Abstract]) AND (agitat*[Title/Abstract] OR delirium [Title/Abstract] OR confus*[Title/Abstract] OR "behavioral change*"[Title/Abstract] OR excitement [Title/Abstract])  #6 #4 OR #5  #7 (randomized controlled trial [Publication Type] OR controlled clinical trial [Publication Type] OR randomized [Title/Abstract] OR placebo [Title/Abstract] OR clinical trials as topic [MeSH: noexp] OR randomly [Title/Abstract] OR trial [Title]) NOT (animals [MeSH Terms] NOT humans [MeSH Terms])  #8 #3 AND #6 AND #7 |
| --- | --- |
| **Cochrane 469** | #1 MESH descriptor Dexmedetomidine explode all trees  #2 Dexmedetomidin* or Precedex or MPV1440 or MPV 1440  or MPV-1440  #3 #1 OR #2  #4 MESH descriptor Emergence Delirium explode all trees  #5 (emergence* or postoperative or postanesthetic) and (agitat* or delirium or confus* or "behavioral change*" or excitement)  #6 #4 OR #5  #7 #3 AND #6 |
| **Embase 320** | #1 'Dexmedetomidine'/exp  #2 (Dexmedetomidin* OR Precedex OR MPV1440 OR  'MPV 1440' OR 'MPV-1440'): ti,ab  #3 #1 OR #2  #4 'Emergence Delirium'/exp  #5 (emergence* OR postoperative OR postanesthetic):ti,ab AND (agitat* OR delirium or confus* OR 'behavioral change' OR excitement):ti,ab  #6 #4 OR #5  #7 'crossover procedure':de OR 'double-blind procedure':de OR 'randomized controlled trial':de OR 'single-blind procedure':de OR (random* OR factorial* OR crossover* OR cross NEXT/1 over* OR placebo* OR doubl* NEAR/1 blind* OR singl* NEAR/1 blind* OR assign* OR allocat* OR volunteer*):de,ab,ti  #8 #3 AND #6 AND #7 |
| **web of science 302** | #1 TS=（Dexmedetomidin* OR Precedex OR MPV1440 OR MPV 1440 OR MPV-1440）  #2 TS= （（emergence* or postoperative OR postanesthetic ）SAME （agitat* OR delirium OR confus* OR "behavioral change*" OR excitement ））  #3 TS= clinical trial* OR TS=research design or TS=comparative stud* OR TS=evaluation stud* OR TS=controlled trial* OR TS=follow-up stud* OR TS=prospective stud* OR TS=random* OR TS=placebo* OR TS=（single blind*）OR TS=（double blind*）  #4 #1 AND #2 AND #3 |
