# Supplementary material for: Effects of Dexmedetomidine on Emergence Agitation and Recovery Quality Among Children Undergoing Surgery Under General Anesthesia: A Meta-Analysis of Randomized Controlled Trials
Source: Front Pediatr. 2020 Nov 13;8:580226. doi: 10.3389/fped.2020.580226 (PMC7694572; doi:10.3389/fped.2020.580226)
Supplement: Supplementary file 1 [file Data_Sheet_1.zip › Supplementary Material/Supplementary Figure legends.doc]

Supplementary Figure legends:

Supplementary Figure 1:Incidence of postoperative nausea and vomiting (Dexmedetomidine vs. control).

Supplementary Figure 2: Number of patients need rescue analgesic (Dexmedetomidine vs. control).

Supplementary Figure 3: Extubation time (Dexmedetomidine vs. control).

Supplementary Figure 4: Emergence time (Dexmedetomidine vs. control).

Supplementary Figure 5: Time to discharge from post anesthesia care unit (Dexmedetomidine vs. control).
